# MicroRNAs and tRNA-Derived Small Fragments: Key Messengers in Nuclear–Mitochondrial Communication

**DOI:** 10.3389/fmolb.2021.643575

**Published:** 2021-05-07

**Authors:** Salvador Meseguer

**Affiliations:** Molecular and Cellular Immunology Laboratory, Centro de Investigación Príncipe Felipe (CIPF), Valencia, Spain

**Keywords:** sncRNA, mt tRF, tRNA fragment, mitochondrial tRNA, microRNA, mitochondrial dysfunction, retrograde signaling, mitochondrial tRNA modification

## Abstract

Mitochondria are not only important as energy suppliers in cells but also participate in other biological processes essential for cell growth and survival. They arose from α-proteobacterial predecessors through endosymbiosis and evolved transferring a large part of their genome to the host cell nucleus. Such a symbiotic relationship has been reinforced over time through increasingly complex signaling mechanisms between the host cell and mitochondria. So far, we do not have a complete view of the mechanisms that allow the mitochondria to communicate their functional status to the nucleus and trigger adaptive and compensatory responses. Recent findings place two classes of small non-coding RNAs (sncRNAs), microRNAs (miRNAs), and tRNA-derived small fragments, in such a scenario, acting as key pieces in the mitochondria–nucleus cross-talk. This review highlights the emerging roles and the interrelation of these sncRNAs in different signaling pathways between mitochondria and the host cell. Moreover, we describe in what way alterations of these complex regulatory mechanisms involving sncRNAs lead to diseases associated with mitochondrial dysfunction. In turn, these discoveries provide novel prognostic biomarker candidates and/or potential therapeutic targets.

## Introduction

Mitochondria are considered the power generators of eukaryotic cells as they are accountable for generating the majority of ATP via the oxidative phosphorylation (OXPHOS) system. Besides ATP production, these organelles serve as fundamental platforms for other cellular processes such as metabolic pathways, cell signaling, and apoptosis (Reinecke et al., 2009; Nunnari and Suomalainen, 2012; Tait and Green, 2012; Chandel, 2014). These mitochondrial activities are essential for cell growth, survival, and function.

Mitochondria harbor its own DNA in a single circular chromosome (mtDNA), which encodes in *Homo sapiens*: 13 intronless, protein-coding genes essential for OXPHOS function and 2 rRNA genes and 22 tRNA genes necessary for the mitochondrial translation machinery. The rest of the OXPHOS subunits, other factors necessary for mitochondrial translation (Suzuki and Suzuki, 2014), besides other proteins crucial for mitochondrial functioning, are encoded in nuclear genes. Because of the dual-compartmental dependence of mitochondrial gene expression, either mtDNA or nDNA mutations can result in the impairment of OXPHOS, a hallmark that is accompanied with highly variable clinical symptoms (Rotig, 2011; DiMauro et al., 2013; Boczonadi and Horvath, 2014). The causes of such extreme variability remain challenging for the scientific community (Reinecke et al., 2009; Chinnery et al., 2012; Nunnari and Suomalainen, 2012; DiMauro et al., 2013; Dogan et al., 2014), although it is increasingly clear that the mitochondria–nucleus communication contributes to the final phenotypic expression (Reinecke et al., 2009; Cagin and Enriquez, 2015). A deep knowledge of this crosstalk is critical for establishing an accurate diagnosis and the development of potential treatments.

Nucleus-to-mitochondria communication (commonly known as anterograde regulation) is orchestrated by a set of transcription factors, nuclear receptors, and cofactors that control the expression of nuclear-encoded mitochondrial genes to adapt mitochondrial activity and biogenesis to the cellular demands (Chandel, 2014; Bohovych and Khalimonchuk, 2016; Quirós et al., 2016). Activity of these downstream effectors is controlled by upstream sensor proteins that perceive variations in the homeostasis of different metabolites [i.e., reactive oxygen species (ROS), Ca^2+^, ADP/ATP, NAD/NADH, etc.] and mitochondrial proteins as consequence of the mitochondrial activity. These regulatory pathways allow the mitochondria to communicate their functional status to the nucleus and are commonly known as retrograde signaling (Chandel, 2014; Bohovych and Khalimonchuk, 2016; Quirós et al., 2016). Recent evidence has highlighted that retrograde signaling plays a part in the clinical phenotype of OXPHOS diseases by turning on adaptive/maladaptive nuclear responses that are alleviated by genetic and epigenetic factors and cell type (Reinecke et al., 2009; Varabyova et al., 2013; Dogan et al., 2014; Picard et al., 2014; Cagin and Enriquez, 2015; Chen et al., 2015).

The involvement of small non-coding RNAs (sncRNAs) in mitochondria–nucleus cross-talk has been poorly investigated. Several studies, some of which have used next-generation sequencing (NGS) strategies, reported the presence of constitutively expressed nuclear-encoded ncRNAs (several tRNAs, 5S rRNA, RMRP, and RNase P) and nuclear- and mitochondrial-encoded sncRNAs and long ncRNAs in mitochondria, providing indications that proteins are not the only mediators of mitochondria homeostasis, but also ncRNAs. However, it is important to mention that these findings are subject to debate. The localization of ncRNAs in the mitochondria is still questioned because of (i) the methodological limitations of separating mitochondria properly from other membrane vesicles (i.e., endoplasmic reticulum), (ii) the detection of small amounts of contaminants by the use of high sensitive NGS techniques, (iii) the lack of independent replicates for most studies by other groups, (iv) the absence of functional characterizations of ncRNAs, and (v) the still unknown transport mechanisms for most ncRNAs.

Despite these controversial aspects, an increasing amount of evidence has linked ncRNAs and their machinery with mitochondria. In this review, we focused on the involvement of sncRNAs in the cross-talk between mitochondria and nucleus in mammals. We consider microRNAs (miRNAs) as many studies have involved them as regulators of mitochondrial function, mainly through directly targeting mRNAs of nuclear-encoded mitochondrial proteins (Vendramin et al., 2017). Moreover, we include an emerging class of regulatory sncRNAs, mitochondrial tRNA fragments (mt tRFs), as they appear to be a novel form of communication from the mitochondria to the nucleus (Meseguer et al., 2019).

microRNAs are ncRNAs of approximately 22 nucleotides (nt) associated with a huge variety of physiological and pathological processes. They are synthesized as primary transcripts (pri-miRNAs) in the nucleus and then processed by the DROSHA/DGCR8 complex into precursor molecules (pre-miRNAs). Exportin 5 (XPO5), along with RanGTP, exports the pre-miRNAs to the cytoplasm, where they are further processed by DICER1 into mature double-stranded miRNAs (Treiber et al., 2018). One strand of mature miRNA, the “passenger strand,” undergoes RNA degradation, whereas the remaining strand, the “guide strand,” associates with the RNA-binding protein argonaute 2 (AGO2), a component of the RNA-induced silencing complex (RISC), to recruit the resulting complex (miRISC) to the target mRNA, by binding to a specific sequence in the 3′ UTR. This miRISC–mRNA interaction results in degradation and/or inhibition of translation of the target mRNA, depending on whether it is mediated by AGO2 and/or GW182, respectively (Carthew and Sontheimer, 2009; Chekulaeva and Filipowicz, 2009; Czech and Hannon, 2011; Iwakawa and Tomari, 2015). In addition, to act as negative posttranscriptional regulators, it has been proposed that miRNA exerts non-canonical functions that occur in other cellular compartments. For instance, miRNAs may act as chromatin and transcriptional regulators in nucleus (i.e., miR-584-3p and miR-26a-1; Liu et al., 2018) and as translational activators in mitochondria (Zhang et al., 2014).

tRNA-derived small fragments (tRFs or tsRNAs) are sncRNAs, approximately 16–35 nt in length, related to a group of physiological and pathological processes such as stemness, cancer, viral infection, immune response, and metabolism (Phizicky and Hopper, 2010; Selitsky et al., 2015; Telonis et al., 2015a; Chiou et al., 2018; Guzzi et al., 2018; Telonis and Rigoutsos, 2018; Meseguer et al., 2019). They originate from the cleavage of precursor or mature tRNAs by specific nucleases under certain conditions including cellular stress (Thompson et al., 2008; Thompson and Parker, 2009; Haussecker et al., 2010; Pederson, 2010; Garcia-Silva et al., 2012; Saikia et al., 2012; Gebetsberger and Polacek, 2013; Wang et al., 2013; Telonis et al., 2015a; Megel et al., 2019). However, constitutive expression has been observed for some tRFs (Honda et al., 2015; Telonis et al., 2015a). They derived from either nuclear- or mitochondrial-encoded tRNA sequences (Ro et al., 2013; Telonis et al., 2015a; Olvedy et al., 2016; Pliatsika et al., 2016, 2018). tRFs resulting from mature tRNAs are classified in six major structural categories depending on their position in relation to the parental tRNA sequence: tRF-5s (also called 5′-tRFs), i-tRFs, tRF-2s, tRF-3s (or 3′-tRFs), 5′-tRNA halves (or 5′-tRHs), and 3′-tRNA halves (or 3′-tRHs) (Pliatsika et al., 2016; Loher et al., 2017; Xie et al., 2020). Two extra categories include tRFs overlapping the precursor tRNA in the 5′ leader (5′U-tRFs) or 3′ trailer (tRF-1s or 3′U-tRFs) sequences. At present, among the RNases involved in tRF biogenesis, we find angiogenin (ANG), RNase T2, Dicer, and RNaseZ/ELAC2. In mammals, investigations have focused on ANG, a member of the RNaseA family, and Dicer, a member of RNase III family (Cole et al., 2009; Ivanov et al., 2011; Diebel et al., 2016). The ribonuclease ANG, activated by stress, cuts mature tRNAs at positions close to the anticodon generating 5′- and 3′-tRHs which, due to their association to this cellular condition, are also known as stress-induced tRHs (Fu et al., 2009; Thompson and Parker, 2009; Ivanov et al., 2011). However, a recent finding showed that only some tRHs depend on ANG, suggesting the participation of yet-to-be-identified enzyme(s) (Su et al., 2019). tRHs disrupt the binding of translation initiation and stability factors within mRNAs and consequently inhibit protein translation (Yamasaki et al., 2009; Zhang et al., 2009; Emara et al., 2010; Ivanov et al., 2011, 2014; Goodarzi et al., 2015). In addition, cytosolic tRHs induce the phospho-eIF2α-independent assembly of stress granules (Emara et al., 2010) and cytoplasmic foci of transiently concentrated untranslated mRNPs (Anderson and Kedersha, 2009). On the other hand, tRFs other than tRHs are produced via either Dicer-dependent (Cole et al., 2009; Yeung et al., 2009; Hasler et al., 2016) or -independent (Li et al., 2012; Kumar et al., 2014) mechanisms. Although the function of tRFs is not yet clear (Pederson, 2010), they can interact with various AGO proteins (Burroughs et al., 2011; Wang et al., 2013; Kuscu et al., 2018) and form biologically active complexes as miRNAs do (Yeung et al., 2009; Kuscu et al., 2018), and therefore, it is thought that tRFs act as negative posttranscriptional regulators of specific mRNAs, although few mRNA targets have been identified so far (Maute et al., 2013; Deng et al., 2015; Huang et al., 2017; Zhou et al., 2017; Kuscu et al., 2018). However, the discovery of interactions between tRFs other than tRHs and the ribosome, leading to translation arrest, or between them and complexes that support translation, has provided more global mechanisms of action for this tRF set (Sobala and Hutvagner, 2013; Keam et al., 2017; Mleczko et al., 2018).

## Influence of miRNAs on Mitochondrial Biology

Based on their genetic origin and cellular location, three classes of mitochondria-related miRNAs can be differentiated: (i) nuclear-encoded miRNAs present in cytoplasm and targeting mitochondria-related transcripts, (ii) nuclear-encoded miRNAs present in mitochondria, and (iii) mtDNA-encoded miRNAs present in mitochondria (Bandiera et al., 2013). The mitochondria-located miRNAs (i and ii) are commonly named as mitomiRs.

### mitomiRs

They originate from either transcription of mtDNA or nuclear genome and subsequent importation to the mitochondria (Barrey et al., 2011; Bandiera et al., 2013). Nuclear-encoded mitomiRs appear to be more abundant in mitochondria than mitochondrial-encoded genes, and their nuclear loci are inside mitochondrial gene clusters or near mitochondrial genes. As expression of miRNA loci and neighboring genes is often coregulated (Baskerville and Bartel, 2005), it has been suggested that a functional connection between both mitomiRs and mitochondrial gene expression could exist.

mitomiRs size and structural and thermodynamic features differ from cytosolic and could facilitate their importation to mitochondria. Most of the proposed import pathways of nucleus-encoded RNAs to mitochondria are largely ATP-dependent (Kim et al., 2017; Jeandard et al., 2019). AGO2, P-bodies, polynucleotide phosphorylase, and voltage-gated ion channels are putative players of these pathways (Salinas et al., 2006; Huang et al., 2011; Zhang et al., 2014; McKenzie et al., 2016; Shepherd et al., 2017). However, evidence that confirms the import of nuclear-encoded miRNAs and the exact mechanism followed for this sncRNA class is still unknown.

Reports on mammalian mitomiRs have been emerging in recent years by using different detection approaches (microarray, reverse transcription-quantitative polymerase chain reaction, and ARS sequencing), as summarized in Table 1. In some cases, both pre-miR and its corresponding mature miRNA were detected in mitochondria, suggesting that miRNA processing may also occur in this organelle (Barrey et al., 2011; Das et al., 2012). In fact, Dicer, responsible for pre-miR processing, has been found in rat brain mitochondria (Wang et al., 2015). However, this finding has not been confirmed for other tissues or species (Das et al., 2012; Ro et al., 2013). Therefore, more studies exploring in more detail this possibility are necessary. Instead, the presence of RISC component AGO2 has been confirmed by different groups suggesting a functional implication of mitomiRs. Nevertheless, the lack of GW182 detection, a factor for miRNA-mediated translational repression, and the small size of 3′ UTRs of mitochondrial mRNAs have led to question if mitomiRs can follow a canonical miRNA activity.

**Table 1:** Examples of miRNAs and their control on mitochondrial biology.

**Name**	**Target(s)**	**Function**	**References**
miR-338	*COX4*	Regulation of oxidative phosphorylation in the axons of sympathetic neurons	Aschrafi et al., 2008
miR-9/9*	*GTPBP3;TRMU/MTO1*	Modulation of mt tRNAs action on mitochondrial translation	Meseguer et al., 2015, 2017
miR-335/335*	*GTPBP3/TRMU*	Modulation of mt tRNAs action on mitochondrial translation	Meseguer et al., 2017
miR-137	*NIX;FUNDC1*	Regulation of mitophagy	Li et al., 2014
miR-210	*COX10;ISCU1/2*	Control of mitochondrial metabolism under hypoxic conditions	Chen et al., 2010
miR-30 family	*P53*	Regulation of mitochondrial fission	Li et al., 2010
miR-1	*IGF-1*	Control of mitochondrial translation and apoptosis	Yu et al., 2008; Zhang et al., 2014
miR-181c	*COXI*	Regulation of oxidative phosphorylation and exercise capacity in rats	Das et al., 2014
miR-23a/b	*GLS*	Regulation of glutamine metabolism	Gao et al., 2009
miR-494	*TFAM;FOXJ3*	Modulation of mitochondrial biogenesis during differentiation of myocytes and adaptation of skeletal muscle to physical exercise	Yamamoto et al., 2012
miR-27a	*GAA;PGM2*	Control of glycolysis	Chemello et al., 2019
miR-142	*FNDC5;IRISIN*	Control of fatty acid oxidation	Chemello et al., 2019
miR-378	*PDK1;CASP9*	Regulation of autophagy and apoptosis in skeletal muscle	Li et al., 2018
miR-2392	*mtDNA(H-strand)*	Control of mtDNA transcription	Fan et al., 2019

*In silico* analyses, using different algorithms, have detected a set of mitomiR seed regions on the mitochondrial genome, which has led scientists to argue the possibility that mitochondrial-encoded transcripts are regulated by mitomiRs. To date, it has been shown for a few mitomiRs that they can posttranslationally regulate mitochondrial-encoded genes, and these findings reported regulation in both directions, either positive or negative (Das et al., 2012, 2014). In particular, systemic administration of miR-181c in rats diminished exercise capacity and caused signs of heart failure, by targeting mitochondrial-encoded COX1 (cytochrome c oxidase subunit 1) (Das et al., 2014). On the contrary, miR-1, highly expressed in mice cardiac and skeletal muscle, recruits ND1 and COX1 mRNAs to mitochondrial ribosomes in AGO2-dependent manner, leading to increased translation (Das et al., 2012). This phenomenon apparently is not due to miRNA recruitment to the 5′-end of their mRNA targets and/or the internal ribosome entry site, both mechanisms implicated in increasing protein synthesis, but the authors argued that a strong interaction between AGO2 and 12S rRNA would bridge the ribosome to the miR-1-bound mitochondrial mRNA, resulting in the stimulated translation. Despite these interesting data, this positive regulation of a mitochondrial mRNA by an mitomiR has not been reported so far for other mitomiRs.

Another opened question is whether mitomiRs could regulate mitochondrial gene expression at other levels rather than posttranscription. In fact, an mitomiR that directly regulates mtDNA transcription has been reported (Fan et al., 2019). In particular, mitomiR-2392 identified target sequences in the H-strand and partially repressed polycistronic mtDNA transcription in a cell-type-specific fashion. This repression also required AGO2 and, as a consequence triggers metabolism reprograming via decrease of OXPHOS and increase of glycolysis that influences chemoresistance in tongue squamous cell carcinoma cells.

Therefore, based on the current information about mitomiRs, they are far from being well known. Not only is a precise identification of mitomiRs in different cell types and species for finding coincident and different elements needed, but also studies to analyze these elements in pathological settings trying to clarify their roles.

### Cytoplasmic miRNAs Targeting Mitochondrial-Related Transcripts

Other miRNAs, despite having not been detected in mitochondria, modulate mitochondrial functions by targeting nuclear-encoded mitochondrial mRNAs in the cytoplasm (Table 1). Frequently, the mitochondrial processes regulated by these miRNAs are OXPHOS, mitochondrial metabolism and dynamics, mitophagy, and apoptosis. For instance, some studies have reported miRNAs affecting one of the primary functions of mitochondria, the ATP synthesis via the OXPHOS system, by altering the expression of OXPHOS subunits. This is the case of a brain-specific miRNA, miR-338, which lowers the expression of the nuclear-encoded cytochrome c oxidase subunit IV (COX4) after its binding to the 3′ UTR of its mRNA (Aschrafi et al., 2008). Other OXPHOS subunits regulated by miRNAs are ATP5B, SDHD, COX10, and CYC (Chen et al., 2010; Puisségur et al., 2011; Zheng et al., 2011; Bukeirat et al., 2016). Although a negative correlation between a miRNA and OXPHOS gene expression is most common, there is also an example of positive regulation by miRNAs, likely through the stabilization of their target mRNAs or activation of their promoters (Carden et al., 2017). In particular, the authors demonstrate in this work that mitochondria dysfunction triggers oxidative stress, which increases methyltransferase activity, resulting in a hypermethylated miR-663 promoter and reduced miR-663 expression. Inhibition of miR-663 expression destabilizes the supercomplexes and reduces OXPHOS enzymatic activities via down-regulation of the expression of nuclear-encoded subunits and assembly factors of respiratory chain complexes I, II, III, and IV, which together contribute to increase tumorigenesis.

miRNAs can also modulate mitochondrial metabolism. As an example, miR-27a-3p and miR-142-3p participate in the regulation of fuel consumption in skeletal muscle (Chemello et al., 2019). A single-myofiber procedure showed opposite expression levels in the intermediate myofibers of miR-27a-3p and miR-142-3p, being highly and lowly expressed, respectively. miR-27a-3p enhances lipid use and reduces the degradation of glycogen and, accordingly, glycolysis in myofibers, whereas miR-142-3p inhibits lipid use. These results reflect the ability of the miRNAs to change the phenotype of a myofiber type from glycolytic to oxidative.

It has also been suggested that some miRNAs participate in the multiple metabolic checkpoints that dictate cell fate (either cell survival or cell death) in response to metabolic fluctuations. A clear example is the metabolic stress-responsive miR-378 that promotes autophagy initiation in the myocytes by targeting the mammalian target of rapamycin/unc-51-like autophagy activating kinase 1 pathway and maintains autophagy via enhancement of Forkhead box class O-mediated transcriptional activity through the repression of phosphoinositide-dependent protein kinase 1 (PDK1) (Li et al., 2018). On the other hand, miR-378 abolishes mitochondrial apoptosis initiation directly by targeting caspase 9, an initiator caspase. Therefore, miR-378 is responsible for integrating metabolic signals into an adaptive response to reestablish homeostasis, reducing the tendency of myocytes to undergo apoptosis by supporting the autophagic process and suppressing apoptotic initiation. Other miRNAs are involved in mitochondrial-mediated apoptosis and are frequently dysregulated in human cancers. For instance, increased levels of miR-21 lead to diminished PTEN expression in human lung and hepatocellular carcinomas (Meng et al., 2007; Zhang et al., 2010), or miRNA-16 and miRNA-15a, which target BCL2, are deleted or underexpressed in chronic lymphocytic leukemia (Cimmino et al., 2005).

Finally, recent studies revealed the engagement of miRNAs in retrograde signaling as a maladaptive response to the OXPHOS dysfunction originated by pathogenic mtDNA mutations (Meseguer et al., 2015, 2017; Wang et al., 2017). On the one hand, Wang et al. showed a set of deregulated muscle miRNAs between MELAS patients with mtDNA A3243G point mutation and controls, which participate in different pathways including the immune system, signal transduction, translation, and muscle contraction. On the other hand, Meseguer and colleagues reported that OXPHOS dysfunction, produced by different pathological mutations, alters the levels of ROS-sensitive miRNAs targeting mt tRNA modification proteins (Meseguer et al., 2015, 2017). By controlling the modification of mt tRNAs, these miRNAs may influence the speed and fidelity of mitochondrial translation to try to palliate the stoichiometric disequilibrium between mtDNA- and nDNA-encoded OXPHOS subunits originated by the pathological mtDNA mutation. However, a persistent alteration of the mt tRNA modification status by miRNAs seems to aggravate the phenotype. A part of miRNAs controlling mt tRNA modification proteins, the same group demonstrated that a pathological mtDNA mutation can produce a deregulation of other miRNAs, which has a considerable effect on nuclear expression and could clarify some traits of the mtDNA disease (Meseguer et al., 2018).

## tRFs, Recently Discovered Regulators of Mitochondria Biology

As mentioned previously, tRFs may originate from both nuclear and mitochondrial tRNAs (Ro et al., 2013; Telonis et al., 2015a,b; Olvedy et al., 2016; Pliatsika et al., 2016, 2018). Based on recent studies, mitochondrial tRFs differ from nuclear tRFs in terms of sequence and size (Telonis et al., 2015a; Loher et al., 2017).

The repetitive representation of tRNA templates in the nuclear genome and the idiosyncrasies of tRNA sequences of tRFs make the determination of tRF profiles from RNA-seq datasets difficult. In this sense, extended regions common among tRNA isodecoders and the existence of plenty of sequences in the nuclear genome matching mitochondrially encoded tRNAs (“mitochondrial tRNA-looklikes”) make the unambiguous establishment of the fragment's source challenging. Furthermore, the human nuclear genome contains hundreds of partial tRNA sequences with lengths approximately half of the mature tRNA length. Only a few sensitive and specific methods like MINTmap (Loher et al., 2017) consider these kinds of difficulties during the mapping of the RNAseq data.

**Figure 1:**
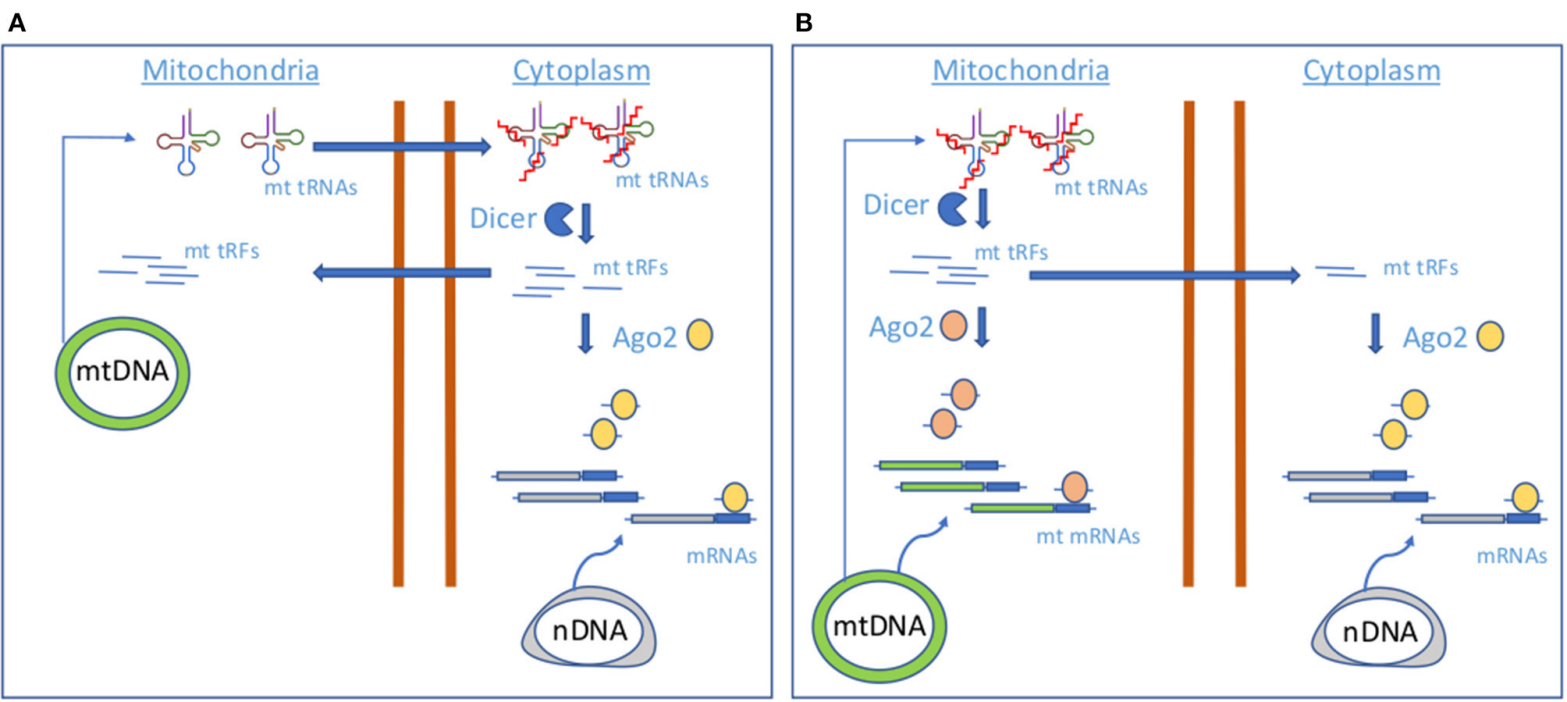
Proposed models of mt tRF biogenesis.**(A)** A model involving cytoplasmic Dicer and AGO2. **(B)** A model involving mitochondrial Dicer and AGO2.

So far, there are few works describing the existence of mitochondrial tRFs and providing evidence about their mitochondrial origin (Loher et al., 2017, 2018; Magee et al., 2018, 2019; Telonis and Rigoutsos, 2018; Meseguer et al., 2019; Londin et al., 2020) and function (Meseguer et al., 2019; Figure 1). Recently, some studies have linked mt tRFs to pathological scenarios. For instance, infection by *Mycobacterium tuberculosis* (Mtb), but not other bacterial pathogens, increases the expression of mitochondria-derived tRFs rather than nucleus-derived tRFs, suggesting a link with mitochondrial distress (Looney et al., 2020). In another study, authors showed that the mitochondrial, internal tRNA-derived RNA fragment, i-tRF-PheGAA, serves as a poor prognosis biomarker in chronic lymphocytic leukemia (Karousi et al., 2020).

On the other hand, Meseguer and collaborators have explored whether the OXPHOS dysfunction produced by a pathogenic mtDNA mutation (m.3243A>G) in the mitochondrial tRNALeu (UUR) gene causing MELAS (mitochondrial encephalomyopathy, lactic acidosis, and stroke-like episodes) altered the production of mt tRFs and, in that case, whether they had a biological function (Meseguer et al., 2019). To test their hypothesis, they used a cybrid model of MELAS. Following a small RNA sequencing and *in silico* analysis using MINTmap, they concluded that the MELAS mutation alters the expression of mt tRFs in comparison to controls. According to MINTmap, they could distinguish between true mt tRFs as their sequences uniquely align to mtDNA-encoded tRNA genes and uncertain mt tRFs because they also match several regions in the nuclear genome. To provide evidence about their real origin, the location of a selected group of mt tRFs within the cell was tested by performing subfraction experiments. They isolated cytoplasm and mitochondrial fractions, which include an RNase treatment to eliminate any RNA anchored to the mitochondrial outer membrane. They found that tRFs derived from mt tRNAs GluUUC, LeuUAA, and ValUAC (mt i-tRF GluUUC, mt 5′-tRF LeuUAA and mt 5′-tRF LeuUAA-m.3243A>G) were preferentially located in mitochondrial fractions but also detected in cytoplasm. In addition, they also probed their mitochondrial origin by evaluating their expression in cells depleted for mtDNA but with the same background as MELAS cells (Rho cells). Their data revealed undetectable levels of mt tRFs in Rho cells.

By silencing the expression of Dicer and AGO2, key components in the miRNA pathway, they found that the levels of certain mt tRFs were diminished. Therefore, they hypothesized that some mt tRFs act as miRNAs and conducted a functional analysis of potential mt tRF targets using a tool for miRNA target prediction. Their analysis revealed a link between mt tRFs and processes involving the most commonly affected tissues in MELAS. They provided evidence of the biological relevance of these fragments, because they demonstrated at least one of them (mt i-tRF GluUUC) regulates one of its predicted targets, the mitochondrial pyruvate carrier 1 (MPC1), promoting the accumulation of extracellular lactate.

Interestingly, they showed that levels of mt i-tRF Glu are dependent on mt tRNA-modifying enzymes operating at wobble uridine position (U34) of the parental mt tRNA, mt tRNA GluUUC (GTPBP3, MTO1, and TRMU). Specifically, they found that MELAS cells exhibited increased levels of mt i-tRF Glu at least in part due to down-regulation of the mt tRNA modification enzymes by the posttranscriptional repressor activity of miR-9/9^*^, a stress-sensitive miRNA. Mechanisms involving hypomodification at key sites of the mature tRNAs and increased sensitivity to nucleases have been reported previously (Schaefer et al., 2010; Blanco et al., 2014; Zhang et al., 2018; Chen et al., 2019), but this study has provided the first case in which the modulation of the modification status of mt tRNAs is controlled by another type of sncRNAs, miRNAs.

The finding of a biological network involving an mt tRF, mt tRNA-modifying enzymes, and a stress-sensitive miRNA could be part of a more general mechanism in which other up- or down-regulated stress-responsive miRNAs in MELAS (Meseguer et al., 2018) may target other enzymes that modify mt tRNAs at other positions. Alterations in the modification status of mt tRNAs may contribute to the build-up of certain mt tRFs that regulate the expression of nuclear genes.

Although mt tRNAs could be dynamically modified as a result of the stress-responsive miRNA action (Meseguer et al., 2015, 2017) and thus control the production of mt tRFs, the molecular players of mt tRF biogenesis remain unknown. Cytosolic tRNA halves production has been attributed to ANG; however, it has not been located in mitochondria (Lyons et al., 2017), whereas the miRNA machinery proteins Dicer and AGO2, putative contributors of cytosolic tRFs generation and function, have been detected in these organelles, although not in all the studies (Das et al., 2012; Ro et al., 2013). As authors found that levels of selected mt tRFs are dependent on proteins Dicer and AGO2, they proposed two mechanisms of mt tRF biogenesis and function based on Dicer and AGO2 localization. In case of a cytosolic Dicer and AGO2 localization (Figure 1A), mt tRNAs would be transported out of the mitochondria, where Dicer would process them in the cytoplasm to produce mt tRFs. Although the transport mechanism is still unknown, the detection of mt tRNAs in the cytoplasm has been previously reported (Maniataki and Mourelatos, 2005). A cytosolic fraction of mt tRFs would be incorporated in AGO2 for the expression control of nuclear-encoded genes, like MPC1. In a mechanism involving mitochondrial Dicer and AGO2 (Figure 1B), Dicer could process mt tRNAs within the mitochondria to produce the mt tRFs that once loaded in mt AGO2 proteins could contribute to regulate the expression of mt DNA-encoded genes. In this line, it has been proposed that mtDNA encodes sncRNAs that may influence mitochondrial gene expression (Ro et al., 2013). However, it could be possible that part of the mitochondrially generated mt tRFs is guided to the cytoplasm for the regulation of nuclear-encoded genes. More studies are necessary to check which of the proposed mechanisms for mt tRF biogenesis is operating.

Finally, mitochondrial-derived tRFs could mediate transgenerational epigenetic inheritance, as has been reported for nuclear-derived tRFs (Chen et al., 2016; Cropley et al., 2016; Sharma et al., 2016). It has been shown that metabolic disorders and behavioral alterations in offspring are the result of paternal environmental conditions (diet or traumatic stress) and the phenotypic transmission occurs via an epigenetic mechanism involving paternal sperm tRFs (Gapp et al., 2014; Rodgers et al., 2015; Chen et al., 2016; Cropley et al., 2016; Sharma et al., 2016; Nätt et al., 2019). Among RNAseq data generated in some of these studies, there are also reads mapping uniquely to mitochondrial tRNA sequences (Gapp et al., 2014; Nätt et al., 2019), which is consistent with the existence of mitochondria in adult sperm (Peña et al., 2009) and the idea of a possible role of paternal mt tRFs in transgenerational epigenetic inheritance. Instead, the possibility of a maternal transgenerational epigenetic inheritance would be supported by the fact that mitochondria are generally transmitted from mother to offspring, so in addition to the transmission of mtDNA, it is tentative to speculate that mt tRFs could also be transferred as maternal epigenetic factors.

## Discussion

The data joined in this brief review point to the fundamental role of mitochondria as signaling platforms through the exchange of ncRNAs between this and other cellular compartments. mtDNA expresses ncRNAs, either in the form of miRNAs or tRFs, which may operate in the nucleus, cytoplasm, or in mitochondria. In turn, mitochondria concentrate nuclear-encoded ncRNAs, acting with this organelle either as a reservoir or facilitating the interaction between these elements and their mitochondrial targets. Likely, the shuttling of ncRNAs might be part of the mitochondria–nucleus crosstalk associated with an energy metabolic state, and a fraction of these elements are responsible for coordinating the balance of the nuclear and mitochondrial transcripts.

A mechanism in which two sncRNAs types, miRNAs and mitochondrial tRFs, are connected through mt tRNA modification and nuclease sensitivity opens a new communication pathway between mitochondria and the nucleus that needs to be investigated in detail in the future. Stress-responsive miRNAs targeting regulators of tRNA cleavage such as mt tRNA-modifying enzymes could be a general scenario in the presence of stress stimuli or in a pathological context. As it has been observed that a main cellular context of tRF production is cellular stress, and miRNAs are involved in many stress responses, the latter could in turn contribute to amplify the stress response through regulation of mt tRF production.

Despite functional pathways or molecular mechanisms involving putative mitochondrial ncRNAs, there are several fundamental questions that remain open. One issue is the transport of ncRNAs through the mitochondria, which requires the crossing of two membranes. A second one is the robustness of the evidence supporting a localization of the ncRNA in a different place rather than the one in which it originated. The existence of mitochondrial sequences in the nuclear genome makes the assessment of a location difficult, and thus experimental validation is necessary. Another question is the evaluation of the regulatory relevance against side products of the expression or processing of other RNAs. And last, determination of biogenesis process for each ncRNA class is also necessary. Obtaining answers to these questions would facilitate the elucidation of the molecular basis of several mitochondrial diseases and the identification of new therapeutic targets.

## Author Contributions

SM wrote the manuscript, elaborated the figure and the table, and approved the review for publication.

## Conflict of Interest

The author declares that the research was conducted in the absence of any commercial or financial relationships that could be construed as a potential conflict of interest.
